# Effectiveness of an educational intervention on the components of the metabolic syndrome of adults with type 2 diabetes: non-randomized clinical trial

**DOI:** 10.17533/udea.iee.v43n1e04

**Published:** 2025-04-15

**Authors:** Wilkslam Alves de Araújo, Isleide Santana Cardoso Santos, Randson Souza Rosa, Cícero Santos Souza, Diego Pires Cruz, Taynnan de Oliveira Damaceno, Tiago Ferreira da Silva Araújo, Gabriela Lemos de Azevedo Maia, Roseanne Montargil Rocha

**Affiliations:** 1 Nurse, Ph.D. Email: wilkslam@hotmail.com. Corresponding author. https://orcid.org/0000-0002-3323-4650 Universidade Estadual do Sudoeste da Bahia Brazil wilkslam@hotmail.com; 2 Nurse, Ph.D. Email: https://orcid.org/0000-0001-8671-8686isantana@uesb.edu.br Universidade Estadual do Sudoeste da Bahia Brazil https://orcid.org/0000-0001-8671-8686isantana@uesb.edu.br; 3 Nurse, Ph.D student. Email: enfrandson@gmail.com https://orcid.org/0000-0001-7093-0578 Universidade Estadual de Feira de Santana Brazil enfrandson@gmail.com; 4 Physician, Graduated in Medical course. Email: cicerossz@hotmail.com https://orcid.org/0000-0001-5715-3583 Universidade Estadual do Sudoeste da Bahia Brazil cicerossz@hotmail.com; 5 Nurse, Ph.D. Email: diego_pcruz@hotmail.com https://orcid.org/0000-0001-9151-9294 Universidade Estadual do Sudoeste da Bahia Brazil diego_pcruz@hotmail.com; 6 Nurse, Master student. Email: dtaynnan@gmail.com https://orcid.org/0000-0002-7507-7593 Universidade Estadual do Sudoeste da Bahia Brazil dtaynnan@gmail.com; 7 Biomedical scientist, Ph.D, Professor. Email: tiago.fsaraujo@univasf.edu.br https://orcid.org/0000-0002-0399-5125 Universidade Federal do Vale do São Francisco Brazil tiago.fsaraujo@univasf.edu.br; 8 Pharmacist, PhD, Professor. Email: gabriela.lam@gmail.com https://orcid.org/0000-0002-6878-4644 Universidade Federal do Vale do São Francisco Brazil gabriela.lam@gmail.com; 9 Nurse, PhD, Professor. Email: rmrocha@uesc.br https://orcid.org/0000-0001-5766-413X Universidade Estadual do Sudoeste da Bahia Brazil rmrocha@uesc.br; 10 State University of Southwest Bahia (SUSB), Jequié, Bahia, Brazil. Universidade Estadual do Sudoeste da Bahia State University of Southwest Bahia (SUSB) Jequié Bahia Brazil; 11 State University of Feira de Santana (UEFS), Feira de Santana/BA, Brazil Universidade Estadual de Feira de Santana State University of Feira de Santana (UEFS) Feira de Santana/BA Brazil; 12 Federal University of Vale do São Francisco (UNIVASF), Petrolina/PE, Brazil Universidade Federal do Vale do São Francisco Federal University of Vale do São Francisco (UNIVASF) Petrolina PE Brazil

**Keywords:** health education, community health nursing, life style, diabetes mellitus type 2, metabolic syndrome., educación en salud, enfermería en salud comunitária, estilo de vida, diabetes mellitus tipo 2, síndrome metabólico., educação em saúde, enfermagem em saúde comunitária, estilo de vida;diabetes mellitus tipo 2, síndrome metabólica.

## Abstract

**Objective.:**

To verify the effectiveness of an educational intervention on the components of metabolic syndrome in adults with type 2 diabetes.

**Methods.:**

A non-randomized clinical trial included 51 adults (48.73±7.84 years old; 86.3% women) diagnosed with type 2 diabetes and metabolic syndrome (intervention group, *n*=26; control group, *n*=25). The intervention consisted of a multidisciplinary health promotion educational program over six months, structured in seven workshops led by nurses. The primary outcome was the improvement of metabolic syndrome components, and the secondary outcome was the reduction in the number of metabolic syndrome criteria assessed at two time points, baseline and after six months of monitoring.

**Results.:**

Compared to the control group, the educational program reduced glucose levels (*p*=0.001) and improved high-density lipoprotein cholesterol concentrations (*p*=0.001) in the intervention group participants at six months. A significant decrease in the mean metabolic syndrome score was observed in the intervention group, while the control group showed an increase (*p*=0.033). At the end of the study, 11.5% of the participants in the intervention group no longer met the criteria for metabolic syndrome.

**Conclusion.:**

A nurse-led health promotion educational program was effective in improving glucose and high-density lipoprotein cholesterol levels among adults with type 2 diabetes and metabolic syndrome, as well as reducing the number of metabolic syndrome components in the participants.

## Resumen

**Objetivo.:**

Verificar la efectividad de una intervención educativa sobre los componentes del síndrome metabólico en adultos con diabetes tipo 2.

**Métodos.:**

Ensayo clínico no aleatorizado que incluyó a 51 adultos (48.73±7.84 años de edad; 86.3% mujeres) diagnosticados con diabetes tipo 2 y síndrome metabólico (grupo de intervención, *n*=26; grupo de control, *n*=25). La intervención consistió en un programa educativo de promoción de la salud de carácter multidisciplinario durante seis meses, estructurado en siete talleres liderados por enfermeros. El desenlace primario fue la mejora de los componentes del síndrome metabólico, y el desenlace secundario fue la reducción en el número de criterios del síndrome metabólico evaluados en dos momentos: en la condición basal y después de seis meses de seguimiento.

**Resultados.:**

En comparación con el grupo de control, el programa educativo redujo los niveles de glucosa (*p*=0.001) y mejoró las concentraciones de colesterol de lipoproteínas de alta densidad (*p*=0.001) en los participantes del grupo de intervención a los seis meses. En el grupo de intervención, se observó una disminución significativa en la puntuación promedio del síndrome metabólico, mientras que el grupo de control mostró un aumento (*p*=0.033). Al final del estudio, el 11.5% de los participantes del grupo de intervención ya no cumplían con los criterios de síndrome metabólico.

**Conclusión.:**

Un programa educativo de promoción de la salud liderado por enfermeros fue eficaz para mejorar los niveles de glucosa y colesterol de lipoproteínas de alta densidad en adultos con diabetes tipo 2 y síndrome metabólico, además de reducir el número de componentes del síndrome metabólico en los participantes.

## Resumo

**Objetivo.:**

Verificar a efetividade de uma intervenção educativa sobre os componentes da síndrome metabólica de adultos com diabetes tipo 2.

**Métodos.:**

Ensaio clínico não-randomizado que incluiu 51 adultos (48.73±7.84 anos de idade; 86.3% mulheres) com diagnóstico de diabetes tipo 2 e síndrome metabólica (grupo intervenção, *n*=26; grupo controle, *n*=25). A intervenção consistiu em um programa educativo de promoção da saúde com caráter multidisciplinar, durante seis meses, estruturado em sete oficinas lideradas por enfermeiros. O desfecho primário foi a melhora dos componentes da síndrome, e o secundário, a redução do número de critérios da síndrome metabólica avaliados em dois momentos, na condição basal e após seis meses de acompanhamento.

**Resultados.:**

Em comparação com o grupo controle, o programa educativo reduziu os níveis de glicose *(p=*0.001) e melhorou as concentrações da lipoproteína-colesterol de alta densidade (*p*=0.001) dos participantes do grupo intervenção aos seis meses. No grupo intervenção, observou-se diminuição significativa na média da pontuação da SM, enquanto o grupo controle apresentou aumento (*p*=0.033). Ao final do estudo 11.5% dos participantes do grupo intervenção não preenchiam mais os critérios de síndrome metabólica.

**Conclusão.:**

Um programa educativo de promoção da saúde liderado por enfermeiros foi eficaz para melhorar os níveis de glicose e da lipoproteína-colesterol de alta densidade entre adultos com diabetes tipo 2 e síndrome metabólica, além de causar redução do número de componentes da síndrome metabólica dos participantes.

## Introduction

The prevalence of metabolic syndrome (MetS) has reached epidemic proportions in the adult population, as well as for the chronic diseases commonly associated with this syndrome, especially type 2 diabetes mellitus (T2DM) and cardiovascular diseases (CVD). This scenario demands one of the greatest concerns in the context of public health worldwide.^1^ It was recently reported that 38.4% of adults have MetS in Brazil, being mainly predominant among women (41.8%) and individuals with a low level of education (47.5%).^2^Considering T2DM cases, the burden of MetS was also generally significantly higher among women (94.43%) with diabetes than among men (76.54%).^3^

Metabolic Syndrome is characterized by a set of cardiometabolic markers that include increased waist circumference, blood glucose, blood pressure, triglycerides and reduced levels of high-density lipoprotein-cholesterol (HDL-c).^4^The association of at least three of these markers defines its diagnosis, and consequently awakens with greater urgency the need for immediate intervention for proper management. MetS causes constant insulin resistance and hyperinsulinemia, which leads to deterioration of β-cell function, so it has often been associated with T2DM.^5^^,^^6^ It was also noticed that high waist circumference, decreased HDL-c and increased blood pressure were the most frequent diagnostic criteria in Brazilian adults diagnosed with MetS.^2^^,^^5^ Also according to the literature, it is a syndrome of complex and multifaceted origin with silent evolution, in addition to not being fully understood.^1^ However, it has been strongly suggested that sedentary lifestyle and unhealthy eating patterns may play a key role in its development. Thus, the chronic nature of DM2, as well as of MetS itself, requires changes in lifestyle and stimulus in the self-care of patients within the scope of primary health care. That is, interventions that mainly consist of increasing the level of physical activity and improving eating habits have been shown to improve the components and risk factors of MetS.^7^^,^^8^ One of our studies also showed improvement in pain domain and increased knowledge about MetS among adults with low education level.^4^Other programs that intervened in lifestyle demonstrated a reduction in the number of diagnostic criteria for MetS.^9^However, most of these programs have an intensive and restrictive character, often focused on weight loss; however, under these conditions, low adherence is the main obstacle.^10^

Currently, there is a lack of data on the effectiveness of health promotion programs aimed at improving knowledge of MetS and the lack of analysis of its effects on the metabolic parameters of adults affected by diabetes and MetS in the context of primary health care, making it difficult to deliver multidisciplinary lifestyle interventions.^11^^,^^12^ Therefore, a multidisciplinary educational program for health promotion was developed, led by nurses, with encouragement for lifestyle changes in adults with MetS. This study aimed to verify the effectiveness of an educational intervention on the components of MetS in adults with DM2.

## Methods

Study design. Study design. This was a non-randomized, open-label, controlled clinical trial with two parallel groups (registration number: RBR-43K52N) conducted in a primary health care center located in the urban area of ​​Jequié, BA, Brazil. This study was part of a larger research project on the evaluation of an educational program in MetS.

Participants. Participants were recruited through an invitation made by the team of researchers during the usual care for hypertension and DM2 in the health center, this initial approach followed a standardized screening protocol for clinical evaluation according to the eligibility criteria of the research. Initially, the sample size was 80 participants, considering an effect size of 0.25, an alpha error of 5%, a statistical power of 80% and a sample loss of 20%.Inclusion criteria included adult (between 18 and 59 years of age) male and female subjects diagnosed with T2DM and three or more of the following criteria for MetS:^13^ (a) waist circumference >102 cm in males and >88 cm in females; (b) fasting glucose ≥100 mg/dl; (c) blood pressure ≥130/85 mmHg; (d) triglycerides ≥150 mg/d and (e) HDL-c <40 mg/dl in males and <50 mg/dl in females. Individuals who presented any of the following situations were excluded: pregnancy and having more than 50% of absences in the workshops. During the clinical screening evaluation, written consent was obtained from all participants.

**Figure 1:**
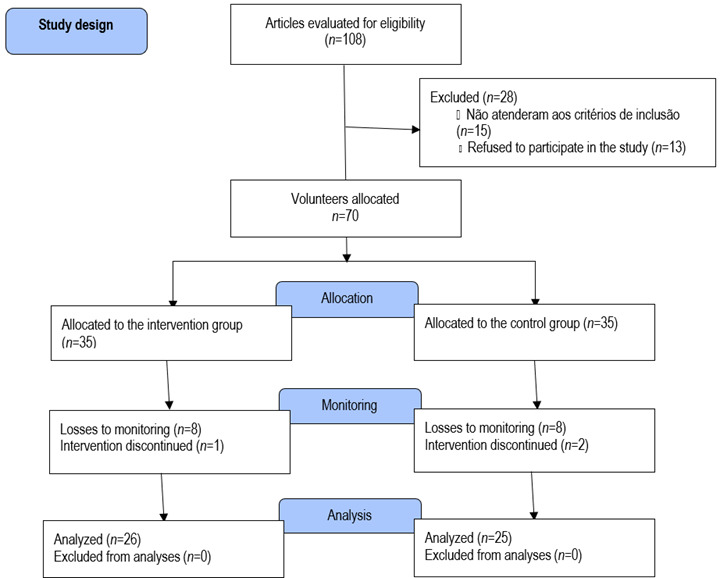
Participant selection flowchart

The distribution flowchart of the study participants is shown in Figure 1. A total of 108 volunteers were recruited, among them 70 adults with diabetes (68.4%) from the health center were considered for analysis of this study. Finally, the eligible participants of the research were intentionally divided into two groups: an intervention group (n=35), which participated in the health promotion educational program entitled "Caring for Educating in the Metabolic Syndrome", and another control group (*n*=35), which maintained usual care. In the intervention group, nine participants were excluded: one for having become pregnant and eight for having low attendance at the workshops. In the control group, ten participants were excluded: two moved to another city and eight had no interest in continuing the research. Therefore, a total of 51 diabetic adults (26 in the intervention group and 25 in the control group) participated in the entire intervention program and were included in the analyses. 

Intervention and control groups. Initially, all study participants received general information about MetS. In addition, each participant individually was informed about the number of MetS components and their condition of high metabolic risk. Participants in the intervention group received, through an educational program, guidance on how to change their lifestyle based on the Pedagogy of Autonomy.^14^Seven group workshops were held, led by nurses, with monthly frequency and duration of 90 to 120 minutes, in the health center itself after routine care. The workshops were structured in two moments, in the first the participants were received, and soon after the project nurses provided knowledge about the aspects of MetS and its risk factors according to the clinical guidelines for adults (concept, diagnosis, treatment, complications and stimuli for behavioral changes).^15^^-^^17^ In the second moment, an invited health professional talked to the participants about topics of interest to the group, which were defined at the end of each workshop. In this multidisciplinary approach, the invited nutritionist provided instructions for maintaining a healthy diet (limiting high-calorie foods and products; paying attention to the quality of fats; increasing the intake of fruits and vegetables, cereals and legumes; daily intake <6 g of iodized salt and reducing alcohol consumption). The psychologist discussed the influence of stress and anxiety as a risk factor for MetS and its associated conditions. We encourage participants to practice at least 150 minutes/week of physical activity; the physical education professional performed some demonstrations of physical activities and exercises. The invited nurse used some integrative practices to work on topics related to the emotions and feelings of the participants. The physical therapist addressed the theme of ergonomics; in addition, he spoke of the importance of stretching and care with posture. The pharmacist discussed the medications and teas used in the components of MetS, and their interactions. Finally, the cardiologist worked on topics related to spirituality and cardiovascular disorders. The nurses who conducted the program, researchers from the Health and Quality of Life Research Group (UESB), received the same instructions and training to collaborate in the execution of the intervention. Participants in the control group did not participate in the educational program and, like the intervention group, maintained the usual care at the health center, with monthly consultation. They received a monthly phone call to confirm their participation in the study, attending the health center to meet the schedule. In addition to the scheduled measurements, there was no other type of personal contact between the researchers and the control group during the study.

Measurements All participants included in the study were evaluated at two time points, before the intervention and after six months of monitoring. The data to characterize the participants were collected at baseline through individual interviews, using a questionnaire structured in three general fields, namely: personal identification (sex, age, color, marital status, years of study and income); general aspects of health (duration of diabetes) and lifestyle (smoking and alcohol use). The level of physical activity was estimated using the International Physical Activity Questionnaire short version (IPAQ). For analysis purposes, participants were classified as physically active (active, irregularly active A and B) and sedentary. MetS was determined using the criteria of the Third Report of the National Cholesterol Education Program Expert Panel on Detection, Evaluation and Treatment of High Blood Cholesterol in Adults (NCEP-ATP III).^13^The abdominal circumference was measured at the midpoint in a horizontal plane between the iliac crest and the lower costal margin, using a flexible and inelastic tape measure with an accuracy of 0.1 cm. Weight was assessed with individuals dressed in light clothing and barefoot, on a portable digital scale (Wiso®, model W801) with a capacity of 0-180 kg and an accuracy of 0.1 kg.^18^Height was measured using a portable metal stadiometer (Sanny, capriche model), with a resolution of 0.1mm. Body mass index (BMI) was obtained using the participant's weight in kilograms divided by the square of their height in meters.^19^Blood pressure was measured with a validated semi-automatic device (Omron, model HEM-742 INT),^20^ with participants in the sitting position, on the left arm, after a ten-minute rest. Systolic and diastolic pressure measurements were represented by the mean of two readings. Blood samples were in the antecubital vein, after confirmation of 12 hours of fasting, in a collection room prepared at the health center. Serum concentrations of triglycerides, HDL-c, and fasting glucose were measured by enzymatic methods (Roche Diagnostics). 

Statistical analysis. We used the general project database, dated March 15, 2020. To report the data, we used mean, standard deviation, frequency and percentage. The normal distribution of data was evaluated by the Shapiro-Wilk test and analysis of homogeneity of variances by the Levene test. To compare variables at baseline between two groups (intervention and control), we used Student's T-test and Chi-square test. All comparisons between groups were based on intent-to-treat analysis using the multiple imputation method. Anova Two-Way (time*group) for repeated measures was used to assess changes in MetS components from baseline to six-month monitoring in all participants, F and p values were reported. To identify the difference pairs, the Bonferroni *post-hoc* was adopted. All statistical analyses were performed by SPSS (version 24.0). The significance level adopted was p<0.05.

Ethical aspects. The ethical approval of the project "Caring for educating in the metabolic syndrome" was obtained by the Research Ethics Committee of the State University of Southwest Bahia (UESB, number CAAE 92352818.9.0000.0055, opinion: 2,850,239). All participants provided written informed consent. 

## Results

Table 1 details the characteristics of the participants at baseline. A total of 51 participants (26 in the intervention group and 25 in the control group) who completed the six-month study were analyzed. Overall, the participants were mostly female (86.3%), middle-aged (48.73±7.84), non-white (78.4%), with a partner (82.4%), with less than eight years of schooling (52.9%), family income of one minimum wage or more (60.8%), never smoked (66.7%) or consumed alcohol (43.1%). Participants reported that they did not practice physical activity (66.7%) and that they have been living for about one to 10 years with the diagnosis of DM2 (60.8%). Based on BMI (34.9 ± 5.9 kg/m^2^), participants in our study ranged from class I to class II obesity (29.4% and 27.5%, respectively). 

**Table 1:** Baseline characteristics of the 51 study participants

Characteristics	**All (*n*=51)**	**Intervention (*n*=26)**	**Control (*n*=25)**
Age_(years)_, mean ± SD	48.73±7.84	48.96±8.03	48.48±7.80
Sex, *n* (%)			
Male	7 (13.7)	5 (19.2)	2 (8.0)
Female	44 (86.3)	21 (80.8)	23 (92.0)
Skin Color, *n* (%)			
White	11 (21.6)	4 (15.4)	7 (28.0)
Non-white	40 (78.4)	22 (84.6)	18 (72.0)
Marital status, *n* (%)			
With partner	42 (82.4)	22 (84.6)	20 (80.0)
Without partner	9 (17.6)	4 (15.4)	5 (20.0)
Years of education, *n* (%)			
< 8 years of schooling	27 (52.9)	15 (57.7)	12 (48.0)
≥ 8 years of schooling	24 (47.1)	11 (42.3)	13 (52.0)
Income, *n* (%)			
< 1 minimum wage	20 (39.2)	12 (46.2)	8 (32.0)
≥ 1 minimum wage	31 (60.8)	14 (53.8)	17 (68.0)
Alcoholic, *n* (%)			
Current	14 (27.5)	7 (26.9)	7 (28.0)
Old	15 (29.4)	6 (23.1)	9 (36.0)
No	22 (43.1)	13 (52.0)	9 (36.0)
Smoker, *n* (%)			
Current	3 (5.9)	2 (7.7)	1 (4.0)
Old	14 (27.5)	6 (23.1)	8 (32.0)
No	34 (66.7)	18 (6.2)	16 (64.0)
Physical activity level, *n* (%)			
Active	17 (33.3)	7 (26.9)	10 (40.0)
Sedentarism	34 (66.7)	19 (73.1)	15 (60.0)
Diabetes duration, *n* (%)			
< 1 year	9 (17.6)	3 (11.5)	6 (24.0)
1 to 10 years	31 (60.8)	19 (73.1)	12 (48.0)
≥ 10 years	11 (21.6)	4 (15.4)	7 (28.0)
Metabolic Syndrome^a^			
MetS score^a^, mean ± SD	4.05±0.75	4.15±0.83	3.96±0.67
Anthropometry, mean ± SD			
Height _(cm)_	156.75±0.81	157.77±0.07	155.68±0.88
Weight _(kg)_	79.50±16.76	79.43±12.90	79.57±20.29
BMI _(kg/m2)_	32.33±6.08	31.86±4.38	32.81±7.53
Abdominal Circumference _(cm)_	105.69±12.73	107.23±9.24	104.08±15.60
Hematology, mean ± SD			
Glucose _(mg/dL)_	174.62±38.97	180.23±38.99	168.80±38.87
Triglycerides _(mg/dL)_	165.80±27.70	169.88±29.30	161.56±25.84
HDL-c_(mg/dL)_	42.02±10.54	39.42±9.38	44.72±11.18
Mean Arterial Pressure ± SD			
Sistolic_(mmHg)_	139.06±17.95	140.04±16.73	138.04±19.43
Diastolic_(mmHg)_	85.33±10.78	85.08±10.62	85.60±11.16

BMI: body mass index. HDL-c: high-density lipoproteins-cholesterol, MetS: metabolic syndrome, ^*^Significantly different from baseline (*p*<0.05)

All subjects had MetS according to NCEP ATP III criteria (mean 4.05±0.75). Both groups had the values of the MetS components changed. No statistically significant difference was detected between groups. Due to the small sample size, it was decided not to perform an analysis between groups stratified by sex, although it recognizes that there are differences between genders in some variables under investigation, such as: waist circumference and HDL-c. The effects of the health promotion educational program on the five metabolic markers are presented in Table 2, from baseline to post-intervention. From the evaluation of the MetS components, a significant trend (*p*=0.001) of group interaction by time for glucose reduction in the intervention group (-33.89 mg/dL) can be verified when compared to the control group (34.00 mg/dL), after the intervention period. We also observed a significant increase (*p*=0.001) in HDL-c interaction for the intervention group (5 mg/dL) compared to the control group (-6.80 mg/dL). No other characteristic changes in MetS were statistically significant.

**Table 2:** Comparisons of MetS components in the control and intervention groups, pre- and post-intervention

Variables	Intervention (n = 26)		Control (n = 25)		ANOVA Time*Group		
	Pre	Post	Pre	Post	F	Right	n_p_^2^
Abdominal Circumference _(cm)_	107.23±9.24	104.15±10.28	104.08±15.60	102.60±1473	0.101	0.752	0.001
Glucose _(mg/dL)_	180.23±38.99	146.34±29.98	168.80±38.87	202.80±37.07	22.177	0.001*	0.185
Triglycerides _(mg/dL)_	163.88±29.30	165.65±36.33	161.56±25.84	164.72±29.07	0.742	0.391	0.008
HDL-c_(mg/dL)_	39.42±9.38	44.42±4.76	44.72±11.18	37.92±8.36	11.370	0.001*	0.106
Systolic blood pressure _(mmHg)_	140.03±16.73	130.23±14.65	138.04±19.43	138.56±19.34	2.190	0.142	0.022
Diastolic blood pressure_, (mmHg)_	85.07±10.62	83.80±9.71	85.60±11.16	85.36±11.69	0.058	0.811	0.001

HDL-c: high-density lipoproteins-cholesterol, ^*^Significantly different from baseline (*p*<0.05)

**Figure 2:**
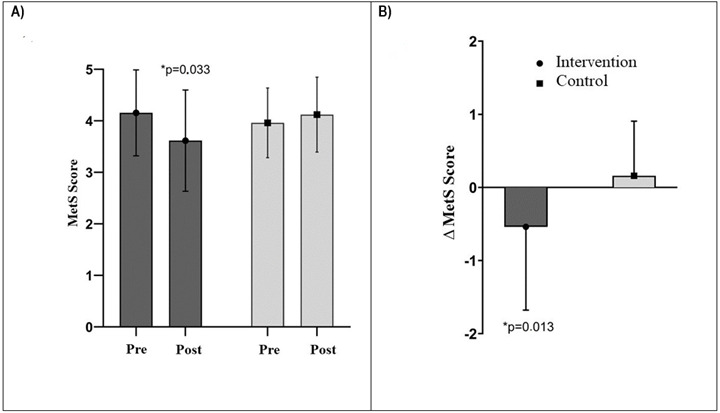
MetS criteria score at baseline and post-intervention

Figure 2 shows the MetS criteria score at baseline and post-intervention. It can be seen that the MetS score revealed a significant interaction effect (*p*=0.033; F= 4.677; n_p_^2^=0.046, Figure 2A) to reduce the mean in the intervention group after the intervention (4.15±0.83 vs. 3.61±0.98), which was not verified in the control group (3.96±0.67 vs. 4.12±0.72) following a trend of increasing the score. It was also found a significant difference in the mean variation of reduction between groups (Δ=-0.53±1.13 intervention group vs. 0.16±0.74 control group, *p*=0.013; Figure 2B). At the end of the study, it was observed that only 11.5% (n=3) of the participants no longer met the MetS criteria. 

## Discussion

This study demonstrated the effectiveness of a multidisciplinary educational intervention program in lifestyle to reduce glucose levels and increase HDL-c concentrations in adults with DM and MetS with low educational level (< 8 years) after six months of implementation, being one of the few interventions led by nurses to promote health in the context of primary health care for the management of MetS. These results are parallel to those of some of the previous studies.^12^^,^^21^ The participation rate in the intervention program was 72.9%, which is a positive indication of its feasibility in clinical practice of a guided lifestyle intervention, which may lead to further improvements in MetS indicators over time. The characteristics of the participants were consistent with previous findings among the adult population with MetS and DM2.^8^^,^^12^ The participants, for the most part, reported that they did not practice physical activity and presented important changes in the MetS markers. Therefore, baseline data indicated the urgent needs for health promotion interventions in this high metabolic risk population. Interestingly, at the end of the study, most participants in the intervention group (in relation to the control group) reported practicing at least 150 minutes of physical activity per week (*n*=20, 60.6% vs *n*=13, 39.4%; ∆=+21.2%).

It is important to highlight the presence and leadership capacity of nursing in terms of coordination and monitoring of the intervention program. Above all, from a multidisciplinary approach perspective, which has been reported as an essential feature in intervention programs aimed at MetS in primary health care.^12^Our research team was concerned with understanding not only the clinical conditions of the participants, but also the aspects of their socio-cultural reality. During the workshops, program professionals acted as support in the care process and provided information to control and improve their MetS clinical condition. Therefore, individuals who received the intervention were stimulated for self-care capacity and to improve knowledge of MetS and its risk factors, as demonstrated in one of our studies.^4^It is also interesting to highlight, in this regard, the presence of nurses who were competent to provide health education on MetS to a majority of people with a low level of education and health knowledge.

The present study indicated a trend of mean reduction in the MetS criteria score after receiving six months of intervention. And the results indicate that regardless of weight loss, lifestyle intervention caused a reduction in mean blood glucose and an increase in HDL-c. However, participants in the control group showed an increase in the mean levels of MetS components from baseline to study completion. A community-based lifestyle intervention study observed a reduction in the prevalence of MetS in overweight women after 16 weeks of intervention of a diabetes prevention program, as well as being able to cause improvements in fasting glucose and HDL-c in those who had high blood glucose and low HDL-c at the beginning of the study.^8^Similarly, other similar studies have also demonstrated significant changes in the total number of MetS components and increased the frequency of individuals achieving resolution of the syndrome.^12^^,^^22^ One program, led by nurses, found a significant reduction in the diagnosis of MetS in the short term by 48.1% (six months) and in the medium term by 83.8% (12 months) in the participants who participated in an interdisciplinary approach intervention with stimulation for physical activity, cognitive behavioral therapy, clinical and nutritional guidance, carried out in a community health center. In contrast to our findings, HDL-c levels increased at 6 and 12 months compared to baseline. Therefore, this finding corroborates a possible cardioprotective effect with the increase in HDL-c levels in those who adhered to the educational intervention program in MetS.^12^This cardioprotective effect was also found by Chang *et al.*,^23^ they observed an increase in HDL-c levels of 2.34 mg/dL at six months. In addition, normalization of glucose metabolism and associated risk factors is crucial for reducing the risk of cardiovascular disease.^24^In our study, on mean, fasting glucose was reduced by 33.89 mg/dL from baseline to the end of the study in the intervention group. It is then suggested that lifestyle modifications may have played a role in lowering blood glucose. This improvement in glycemic profile was similar to previous studies.^12^^,^^21^ For example, fasting glucose was significantly reduced in the group that participated in a lifestyle modification program, with a significant decrease in the prevalence of hyperglycemia in 38.4% of cases.^21^

These metabolic benefits are likely to end up not only resulting in a lower incidence of MetS, but also reducing cardiovascular events in these patients with DM and MetS. This result, which is in line with the improvement in MetS criteria, may also reflect an important reduction in cardiovascular risk. It was found that the results obtained coincide with other studies that show the effectiveness of health education for cardiometabolic health.^21^^,^^24^ It has been shown in the literature that the risk of developing complications among adults with T2DM was significantly lower in those participants who decreased glucose levels.^25^Interestingly, another study demonstrated that the risk remained significantly reduced, even if the reversion to normal glucose levels was only transient.^24^

In a current study, participants were encouraged to incorporate regular physical activity and healthier eating habits with recommendations for self-management of daily fat and calorie intake.^4^Other evidence also highlights the benefits of these recommendations for improving the chances of getting rid of MetS.^8^^,^^26^ Thus, a possible explanation may be due to the changes that the participants themselves underwent towards a healthy lifestyle, which agrees with a study that reported that the therapeutic approach to lifestyle can benefit an improvement in glycemia and HDL-c.^7^^,^^8^^,^^11^ However, we cannot say whether the participants decreased their caloric intake in relation to baseline values. Physical activity is widely described in the literature as one of the main protective factors against various chronic diseases, including T2DM and MetS^6^ Indeed, a meta-analysis showed that regular practice of physical activity, both supervised and unsupervised, could decrease serum glucose levels among patients with T2DM.^25^^,^^27^ It has also been associated with improvements in the anti-inflammatory properties of HDL-c.^28^Elevated HDL-c levels have been linked to lower risks of cardiovascular disease.^28^In agreement with the present study, a previous study demonstrated improvement in HDL-c after a change in lifestyle.^12^However, even with improvements in blood glucose and HDL-c levels, the values still remained altered at the end of the study according to the definitions of NCEP-ATP III. Thus, it is suggested that studies be carried out with longer duration and monitoring. On the other hand, this improvement, even if still at altered levels, is important in relation to the efficiency of the intervention program. Since a cross-sectional study, it was observed that women with diabetes were more likely to have obesity and reduced HDL-c levels.^3^

On the other hand, although there was only a modest increase in HDL-c from baseline to the end of the study (at six months), the results of the analysis suggested that the control group experienced a reduction in HDL-c. In addition, regardless of the magnitude of the reduction, the existing evidence was consistent with the present study in terms of changes in HDL-c^12^ For example, in the study by Rodríguez *et al.*^7^ an educational intervention was carried out with women (n= 230, 53.16±4.30 years of age) from two health centers, who were premenopausal and with at least one cardiovascular risk factor. After one-year monitoring, women in the intervention group had better HDL-_C_ and glucose levels compared to women in the control group. The consequences of these interactions contributed significantly to a decrease in the occurrence of MetS and, inevitably, to a decrease in the risk of CVD. On the other hand, no significant effects of the intervention on waist circumference, triglycerides and blood pressure were observed. Although the changes were not significant, blood pressure and waist circumference improved somewhat during the intervention period. To observe significant effects on these variables, an intervention period longer than the six months performed in the present study may be necessary. Further studies are needed to determine whether such effects can be observed for a longer period.

These results show that the prevention of the components of MetS in adults with T2DM is an important activity to be carried out by health professionals within the scope of primary health care and that the adult population seems to be receptive to these initiatives. In the specific case of adults with multimorbidities, the need for preventive actions becomes much more evident, given the greater possibility of developing CVD. Especially when one observes the trend of increasing prevalence of T2DM and MetS in the adult Brazilian population in general and particularly in women.^3^Therefore, this evidence suggests that the implementation of intervention programs to improve evidence-based lifestyle at the primary health care level may attenuate the progression of MetS and, consequently, the long-term burden of chronic diseases.

However, we believe that the results of this study, although incipient, emphasize the value of health promotion aimed at the management of MetS and contribute as evidence to support future policies that require a greater focus on prevention in primary care of the adult population at cardiometabolic risk. Thus, this study highlights the clinical value of an educational program for MetS, with seven group workshops versus the usual care in a public health center for diabetic adults with MetS. Especially, considering the predominance of women in the study, as well as the age group they are in, it is worth noting that several studies demonstrate that the cessation of menstruation causes an increased cardiovascular risk in women, due to the deficiency of estrogen, a cardioprotective hormone. In addition, in this phase, metabolic changes occur, such as increased blood pressure, triglycerides and decreased HDL-c. ^7^ On the other hand, among the limitations, we must highlight that the main limitation of this study was the small sample size and lack of randomization of participants to the intervention or control group. However, random distribution was unfeasible due to the specificity of the eligibility criteria, as well as the participants' own family and professional needs. Blinding was also not possible, since all participants belong to the same community and the same health center. In any case, this approach seems to be important, as it reflects the real-life condition of users in the health system. However, it may limit the generalization of the results and, therefore, more studies are needed to consider these aspects. The intervention period was limited (six months). We believe that determining the effectiveness of the intervention over a longer period and including more health centers would be necessary. In addition, our overall results are clinically significant and certainly support the role of lifestyle interventions in diabetic individuals with MetS, under nursing monitoring and by specialists for six months.

Therefore, the findings of this study can be used to support the application of evidence in community-level public health programs to improve glucose and HLD-c in adults, especially women, with DM and MetS. This finding is relevant, as there is a need to consolidate the implementation of educational programs for intervention in MetS at the level of primary health care. Thus, we suggest that programs to encourage healthier lifestyle habits that include an interdisciplinary approach with nursing leadership should be promoted with the population as a way to improve the lipid profile and prevent or reverse insulin resistance, as well as prevent chronic diseases. These findings highlight the importance of nursing leadership in the context of primary health care in the management of MetS and T2DM. The involvement of nurses in educational programs for these conditions demonstrates their ability to lead interventions and identify early diagnostic criteria for MetS.^29^The promotion of self-care and adherence to lifestyle changes, such as the practice of physical activity and the adoption of healthy eating habits, are reinforced by health education, regardless of the level of education of the participants. Thus, nursing plays an important role in the prevention and progression of both MetS and T2DM. ^30^

Although longer monitoring periods are needed for a more robust assessment, the results of this study reinforce the importance of patient-centered care strategies, with a focus on early screening, ongoing monitoring, and educational interventions. Nursing participation is essential in the multidisciplinary care of adults with MetS and T2DM, contributing to reduce the burden of chronic diseases in vulnerable populations. The role of nurses in educational interventions is essential to promote adherence to lifestyle changes. In addition, multidisciplinary interventions, especially in individuals with multimorbidities, have a better prognosis when there is joint planning among health professionals. Therefore, the development of actions by nursing in primary health care promotes the prevention of chronic conditions and improves the quality of life of these people, who are often unaware of MetS and face challenges in the management of T2DM. ^30^ The great contribution of this study to nursing as a science lies in demonstrating its essential role in health promotion and in the management of chronic conditions, such as MetS and T2DM. By integrating technical knowledge, intervention planning and health education nursing is consolidated as a scientific discipline capable of leading effective care strategies, directly impacting the strengthening of public health practices.

Conclusion. The intervention of an educational program led by nurses, to promote health in lifestyle caused a reduction in fasting blood glucose and improved HDL-_C_ concentrations in adults with DM and MetS with low educational level, after six months of group intervention. Our findings suggest that the investigated program may have a significant impact in reducing the number of MetS components in the context of primary health care.
